# Cadmium-Sensitive Measurement Using a Nano-Copper-Enhanced Carbon Fiber Electrode

**DOI:** 10.3390/s19224901

**Published:** 2019-11-09

**Authors:** Jian Wu, Zhipeng Xu, Xian Wang, Li Wang, Huadong Qiu, Kechao Lu, Wenhong Zhang, Qing Feng, Jun Chen, Lei Yang

**Affiliations:** 1 School of Mechanical & Automotive Engineering, Qilu University of Technology (Shandong Academy of Sciences), Jinan 250353, China; 1043118197@stu.qlu.edu.cn (J.W.); 15275188150@163.com (H.Q.); lukechao178@163.com (K.L.); whzpcx@163.com (W.Z.); 2 Jinan Foreign Language School, Jinan 250014, China; xzp020619@163.com; 3 Department of Mechanical & Industrial Engineering, University of Toronto, 5 King’s College Rd, Toronto, Ontario M5S 3G8, Canada; xianjeremy.wang@mail.utoronto.ca; 4 School of Environmental Science & Engineering, Qilu University of Technology (Shandong Academy of Sciences), Jinan 250353, China; qingfeng@qlu.edu.cn; 5 College of information science and engineering, Shandong Agricultural University, Tai’an 271018, China

**Keywords:** cadmium ion, carbon fiber electrode, nano-copper, sensitivity for cadmium ion detection, charge transferability

## Abstract

Enrichment of cadmium ion (Cd^2+^) from the environment may lead to kidney disease and weakened immunity in the body. Current techniques are not convenient enough to measure Cd^2+^ concentration in the environment due to low sensitivity and poor linear range. In this paper, a new measurement technique is proposed using a new sensing electrode made of nano-copper-enhanced carbon fiber. Nano-copper was deposited onto the surface of carbon fiber to enhance the current concentration and mass transfer rate of Cd^2+^ during measurement, which improved the electrochemical detection sensitivity significantly (by up to 3.7 × 10^8^ nA/nM) and broadened the linear range to 10~10^5^ nM. This device provides a low-cost solution for measuring Cd^2+^ concentration in the environment.

## 1. Introduction

Heavy metal ions such as cadmium ions (Cd^2+^) are highly toxic to human beings [1]. Cd^2+^ enrichment in human organs like kidney and bone can cause malfunctions in the metabolism of calcium and urine protein, and possibly lead to osteocarcinoma and kidney failure. In addition, cadmium is one of the major environmental pollutants in agricultural production and sanitation [2,3,4,5].

In the 1940s, serious cadmium-pollution-induced osteocarcinoma in Japan raised public awareness of cadmium’s toxic effects for the first time [6,7]. Recently, another cadmium contamination event in the Longjiang River caused 2.8 million fish deaths and serious threats to human health nearby [8]. If a large number of people in an area get sick or die due to the contamination of cadmium, it is necessary to consider whether the cadmium concentration in the area is over the acceptable limit. Accordingly, a convenient method for measuring cadmium concentration is in great need. 

To achieve this goal, many measuring methods for heavy metal ions have been developed [9]. Terahertz spectroscopy is one of the advanced techniques used. The sample is scanned by terahertz spectroscopy using electromagnetic radiation (0.1 THz to 10 THz) and characteristic absorption peaks appear at corresponding frequencies in the spectrum [10,11,12]. However, this technique is often used to measure caesium ions [13]. In fact, many different kinds of metal ions, including Cd^2+^, can cause a detrimental effect on the human body, which is out of the detectable range of elements in terahertz spectroscopy [14,15]. D’Auria’s group invented a protein-bounded biosensor for cadmium detection. It achieved a high sensitivity of 2.5 µg/mL ~ 10 µg/mL and a lower detection limit of 0.5 µM. However, its performance highly depends on the temperature and pH conditions because the sensing part is a protein, i.e., metallothionein (MT) [16].

In recent years, electrochemical methods have become popular in detection of heavy metal ions due to advantages such as high sensitivity, simple procedures, and short analysis time [17]. Aswathi developed a sensor by depositing MoS_2_ onto a glassy carbon electrode (GCE) substrate, which achieved a detection performance of Cd^2+^ of 1.0 × 10^–12^ mol/L [18]. However, the contact area of the GCE (diameter = 3~5 mm) was much larger than that of the micro-electrode, which was made of carbon fiber and had a diameter of 8 μm [19]. This issue may reduce the analyte diffusion rate of the electrodes. As a result, GCE is not suitable for fast measurement of transient electrochemical reactions [20]. 

Recently, microelectrodes have been used for measuring heavy metal ions including Cd^2+^ [21]. For example, a gold-based microelectrode fabricated by microelectromechanical systems (MEMS) technology was used to measure Cd^2+^ in serum and achieved a sensitivity of 3.93 μA/nM [22]. A 64-microelectrode array can simultaneously measure Cd^2+^ and Hg^2+^ and reach a sensitivity of 28 nA/nM [23]. A new hybrid nanocomposite microelectrode was composed of one-dimensional multi-walled carbon nanotubes (MWCNTs) and two-dimensional graphene oxide flakes. This microelectrode improved the linear calibration range for Pb^2+^ and Cd^2+^ to 0.5–30 μg/L and the detection limits for Cd^2+^ to 0.61 nM (signal-to-noise ratio = 3, which is a threshold for evaluating effectiveness of the measured signal) [24]. Nanocomposite modification on electrode may improve the response time, sensitivity, and especially the linear sensing range and detection limit in Cd^2+^ measurement. Because the number of active electrons on the surface of the microelectrode could be increased and more chances to contact analytes would be created for electrochemical reactions [25]. 

In this paper, a carbon fiber modified with nano-copper deposition is used for Cd^2+^ detection. Optimal deposition times were tested experimentally and the results showed that a deposition time of 80 s could achieve the best sensitivity. In addition, the influence of pH was explored. The detection limit of the microelectrode after modification with nano-copper was improved to 10 nM. The sensitivity of the microelectrode was improved to 3.7 × 108 nA/nM. Finally, the proposed sensor was used to test water samples from a river, lake, and running water. 

## 2. Experimental Methods 

### 2.1. Fabrication of the Carbon Fiber Electrode

**Figure 1:**
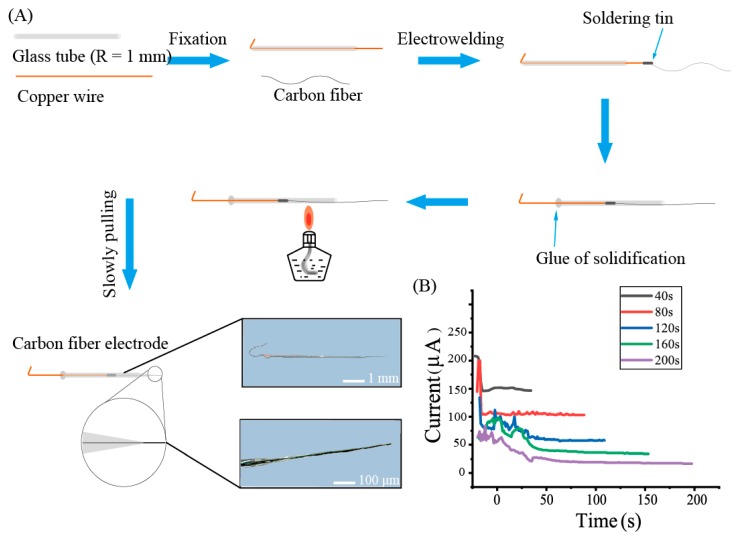
(**A**) A schematic for demonstrating each step during the fabrication process of the carbon fiber electrode. The digital images show a fabricated carbon fiber electrode and its tip shape. (**B**) The current change during the deposition of the nano-copper onto the surface of the carbon fiber.

The preparation process of carbon fiber electrode is described as follows (Figure 1A): (1) A copper wire with a diameter of 100 μm and a length of 70 mm was inserted into a glass tube (inner diameter = 1.1 mm). (2) The glass tube was fixed by a holding device in the micromanipulator and the copper wire was welded to the carbon fiber using an electric soldering iron. The welded copper wire and carbon fiber were then moved to the centre of the glass tube. (3) The glass tube was calcined by the outer flame of the alcohol lamp. When the tube centre was melted by the flame, the glass tube broke up into two parts due to a preload force applied on its two terminals. (4) The carbon fiber was surrounded in the glass tube and formed a carbon fiber electrode.

### 2.2. Nano-Copper Deposition and Electrochemical Measurements

To enhance the electrochemical performance (e.g., sensitivity) of the carbon fiber microelectrode, nano-copper was deposited onto the surface of the carbon fibers by a potentiostatic method under different reaction conditions. First, a carbon fiber was sequentially washed using acetone, isopropanol and distilled water. After drying at 80 °C, the carbon fiber was cleaned using plasma for 30 s at the power of 50 W. CuSO_4_ (0.3 g) was dissolved in 20 mL of supporting electrolyte (0.9% NaCl). The potentiostatic method was applied on an electrochemical work station (Metrohm-Autolab PGSTAT302N, Metrohm Autolab B.V., Utrecht, The Netherlands). A potential of 7.5 V was applied to the carbon fiber at room temperature. Six groups of reaction conditions (depositing time = 40 s, 80 s, 120 s, 160 s, and 200 s) were performed for the same type of carbon fibers, which aims to locate the proper depositing time for the optimal sensitivity of carbon fiber. After electrodeposition, the modified carbon fibers were washed with ethanol and distilled water in sequence to remove all loosely-bound nano-copper particles, and then dried in a vacuum at room temperature. 

During measurement, the prepared electrode was placed in an electrochemical cell containing the analyte solution. The performance of the prepared electrochemical sensors was tested using analytical grade chemicals including CdCl_2_, CuSO_4_, KCl, HCl, and NaOH, etc. Differential pulse voltammetry (DPV) and electrochemical impedance spectroscopy (EIS) were carried out on the electrochemical workstation (Metrohm-Autolab PGSTAT302N). The data were processed by using the softwares FRA and NOVA 1.11. The three-electrode system was used in all electrochemical experiments. NaCl solution (0.9%) was used as the supporting solution. Two groups of electrodes including carbon fiber modified with and without nano-copper were used as the working electrode. Ag/AgCl and platinum wires were used as the reference electrode and the counter electrode, respectively. Before each test, the surface of the carbon fiber was washed by cyclic voltammetry (CV) scanning for 300 cycles in a 0.9% NaCl solution. 

## 3. Results and Discussion

### 3.1. Exploration of Nano-Copper Modification Conditions on the Surface of Carbon Fiber

#### 3.1.1. Modification of Nano-Copper on Carbon Fiber

To observe the optimal performance of nano-copper, different depositing times (including 40 s, 80 s, 120 s, 160 s, and 200 s) were explored. The anode current with different depositing times were recorded and plotted in Figure 1B, which showed that the anode current decreased from 150 μA to 20 μA at the end of each group. This indicates that the resistance decreased during the process of electrodeposition due to the accumulation of free electrons [26]. 

**Figure 2:**
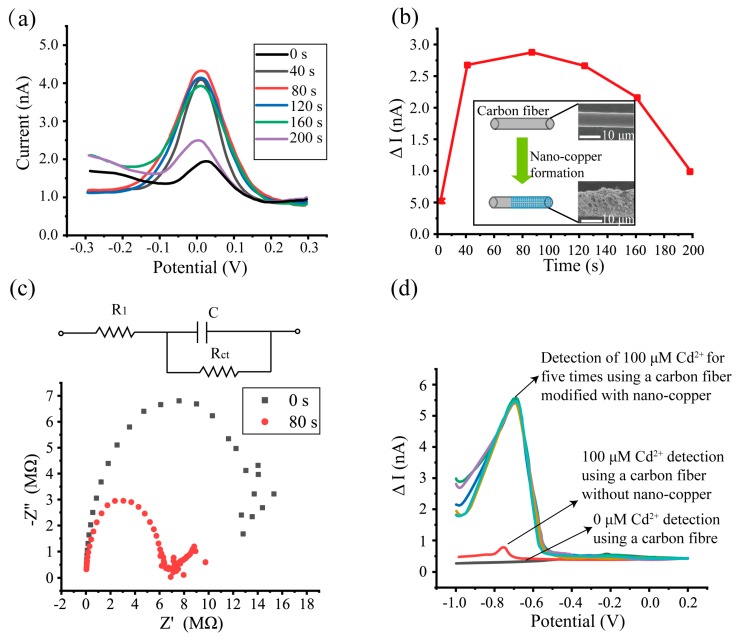
(**a**) Exploration of nano-copper modification conditions. Differential pulse voltammetry (DPV) responses of 5 mM ${{\left[\mathrm{Fe}(\mathrm{CN}\right)}_{6}]}^{3-}$ using carbon fibers modified with different deposition times. (**b**) The measured ΔI for different deposition times (ΔI was the largest with a deposition time of 80 s). The SEM images of carbon fiber with and without nano-copper show significant surface morphology changes. (**c**) Verification of charge transferability by the electrochemical impedance spectroscopy (EIS) response for the carbon fibers with and without nano-copper (80 s and 0 s, respectively). (**d**) DPV responses for 100 μM Cd^2+^ solution and supporting solution without Cd^2+^.

To determine the optimal electrodeposition condition, six groups of carbon fiber electrodes including a control group (the carbon fiber without nano-copper) were immersed in [Fe(CN)_6_]^3−^ to test their DPV response. [Fe(CN)_6_]^3−^ is commonly used as a bentchmark for evaluating the performance of electrochemical sensors. Figure 2a shows the DPV response of carbon fiber with and without nano-copper for [Fe(CN)_6_]^3−^. The oxidation peak of the carbon fiber without nano-copper occurs at a potential of 35 mV and its oxidation peak current is 1.96 nA. The difference between the peak current and background current is defined as ΔI, which refers to the first point of Figure 2b. The oxidation peak current dramatically increases to 4.1 nA for the carbon fiber with nano-copper that was deposited under 40 s, and the oxidation peak shifts to 7 mV. When the deposition time is increased to 80 s, the oxidation peak current changes to 4.3 nA. However, the oxidation peak current decreases with the increase in deposition time, and its value decreases to 2.5 nA (ΔI is 0.92 nA, shown in the sixth point of Figure 2b). From the DPV responses in the [Fe(CN)_6_]^3−^ experiment, 80 s was selected as the deposition time for nano-copper modification.

#### 3.1.2. Characterization of Charge Transferability 

Electrochemical impedance spectroscopy (EIS) was adopted to verify the difference in charge transferability between the carbon fibers with and without nano-copper. Figure 2c shows Nyquist plots of the carbon fibers that were scanned in 5 mM [Fe(CN)_6_]^3+^. The appearance of a semicircle in a Nyquist plot indicates that in the impedance model (see the inset in Figure 2c) there is a parallel connection between the resistor R_ct_ (charge transfer resistance) and the capacitor C_del_ (double electric layer). The calculated R_ct_ in carbon fiber without nano-copper was 17.3 kΩ and that for carbon fiber with nano-copper was 6.5 kΩ. A significant decrease (62.4%) in R_ct_ reveals that the nano-copper deposition is able to increase the electron transfer speed and enhance diffusion of reactive chemical species.

#### 3.1.3. Morphologies of Carbon Fibers with and without Nano-Copper

To determine the mechanism of how the nano-copper deposition is able to enhance electrochemical sensing performance, the morphology change of carbon fiber before and after nano-copper modification was studied by SEM (S4800 microscope, Hitachi, Ltd. Hitachi, Japan). The inset in Figure 2b shows that carbon fiber without nano-copper has a smooth cylindrical surface, and its diameter is 7 μm. After depositing for 80 s, rough nano-copper was evenly grown onto the surface of the carbon fiber and the diameter of the modified carbon fiber increased to 8.5 μm. The increased diameter and the roughness of the surface improved the specific surface area significantly, which provided more reaction sites for the bounding of the electrode and Cd^2+^. 

### 3.2. DPV Response Comparison of the Carbon Fiber Electrode with and without Nano-Copper

To compare the redox response of Cd^2+^ for the carbon fiber electrodes with and without nano-copper, DPV was employed to test a 100 μM Cd^2+^ solution group and another control group of solution without Cd^2+^. There was no oxidation peak (redox response did not occur) when carbon fiber electrodes were tested in the control group of solution without Cd^2+^. For the carbon fiber electrode without nano-copper, there was an oxidation peak at a potential of –751 mV with a value of 0.82 nA. However, the oxidation peak current increased to 5.6 nA for the carbon fiber electrode with nano-copper and the potential of the oxidation peak shifted to –696 mV. The significant improvement of the DPV response may be attributed to the enlarged specific surface area and more reaction contacting spots after nano-copper deposition [27].

In addition, the DPV measurement for the carbon fiber with nano-copper was repeated for five times to test its stability. It can be seen from Figure 2d that the peak current stabilized around 5.6 nA. This indicates that the electrode can achieve a steady response output for Cd^2+^ detection.

### 3.3. The Influence of pH

**Figure 3:**
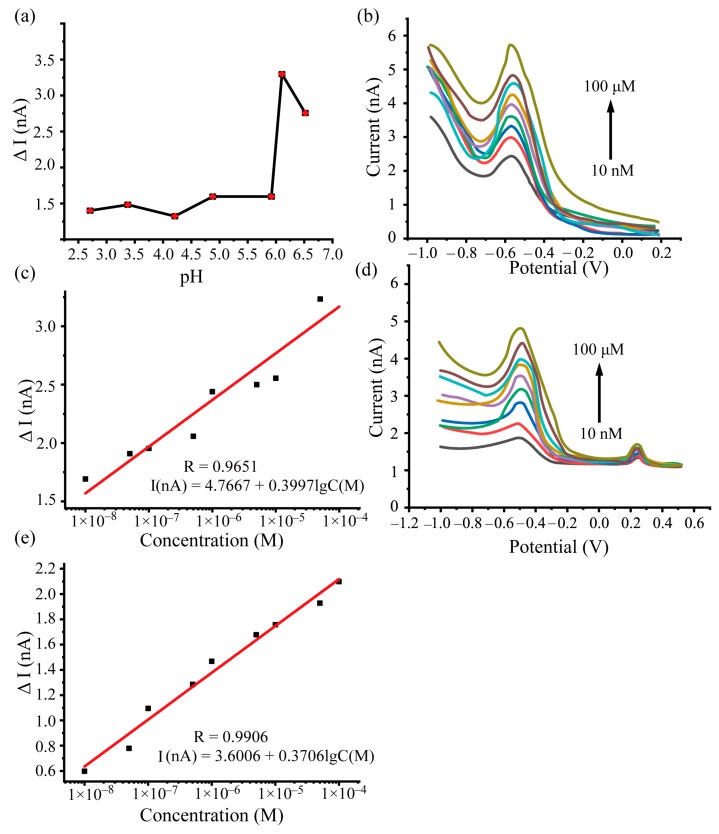
(**a**) The influence of pH in the DPV response for Cd^2+^. The pH was changed from 2.7 to 6.6. (**b**) DPV responses of nano-copper-modified carbon fiber with different concentrations of Cd^2+^ from 10 nM to 100 μM. (**c**) The fitting result between ΔI (the difference between the peak current and background current) and the concentration of Cd^2+^; ΔI = k lg(C) + b. (**d**) The DPV responses for the mixing of foreign ions including K^+^, Na^+^, Cu^2+^, Hg^2+^, Al^3+^, (SO_4_)^2-^, and (NO_3_)^-^. (**e**) The fitting result between ΔI and the concentration of Cd^2+^ in the presence of foreign ions.

It is well accepted that the pH of the aqueous solution would affect the electrocatalytic reaction in the electrochemical redox process [28]. Thus, the influence of pH on detecting Cd^2+^ was investigated for the carbon-fiber-modified electrode (Figure 3a). The pH changed from 2.7 to 6.6 and the ΔI was used to compare the influence of pH. The ΔI slowly increased when the pH changed from 2.7 to 5.9. When the pH achieved a value of 6.1, the ΔI increased to the maximum level (3.25 nA). After this, the ΔI started to decrease. Therefore, pH = 6.1 was selected for testing Cd^2+^.

### 3.4. Determination of the Sensitivity, Detection Limit, and Linear Range for Cd^2+^ Measurement

To evaluate the performance including the linear range, sensitivity and detection limit of the proposed carbon fiber electrode, Cd^2+^ solutions from 10 nM to 100 μM was used as the testing sample. Figure 3b shows that the oxidation current increases with the concentration of Cd^2+^. When Cd^2+^ concentration is less than 1 nM and 5 nM in the supporting solution, there is no detectable current response. When the Cd^2+^ concentration is increased to 10 nM, an oxidation peak was detected (signal-to-noise ratio > 3) which is referred as the detection limit of the carbon fiber electrode for Cd^2+^. The specific values of oxidized peak currents were evaluated and were linear fitted with the log concentration of Cd^2+^ in the form ΔI = k lg(C) + b. The fitting result (see Figure 3c) shows that the sensitivity of carbon fiber with nano-copper for Cd^2+^ is 0.3997 nA/nM and that the linear range is the whole detection range (10 nM to 100 μM). In addition, the Cd^2+^ sensing performance of our nano-copper-modified carbon fiber electrode and other electrodes in the literature are compared in Table 1, which shows that the proposed electrodes have superiority in the detection limit and the linear range. 

### 3.5. The Selectivity of the Carbon Fiber Electrode with Nano-Copper

To determine the selectivity of our electrode, the DPV response was tested in the supporting solution with foreign ions, including 500 μM K^+^, Na^+^, Cu^2+^, Hg^2+^, Al^3+^, (SO_4_)^2–^, and (NO_3_)^–^. The amount of foreign ions was five-fold to 50,000-fold that of Cd^2+^. Figure 3d shows the DPV response for the mixed solutions. During the voltage scanning range (from –1.1 V to 0.5 V), obvious oxidation peaks occur around a potential of 224 mV. This is probably due to the presence of Hg^2+^, according to the reported results in the literature [26,40]. 

There is also an inconspicuous peak around the potential of –300 mV, which may be the response of Cu^2+^. However, the peak is mostly covered by the DPV response of Cd^2+^. The results reveal that foreign ions have little effect on Cd^2+^ detection.

In addition, it was evaluated whether the foreign ions affect the linear range, sensitivity, and detection limit of the proposed carbon fiber electrode. The curve between Cd^2+^ concentration and DPV peak current was fitted (see Figure 3e). This indicated that the linear range and the detection limit did not change when the interference was presented while the sensitivity was slightly decreased by 7.28%. 

### 3.6. Analytical Application to Water Samples Collected from Water Sources

**Figure 4:**
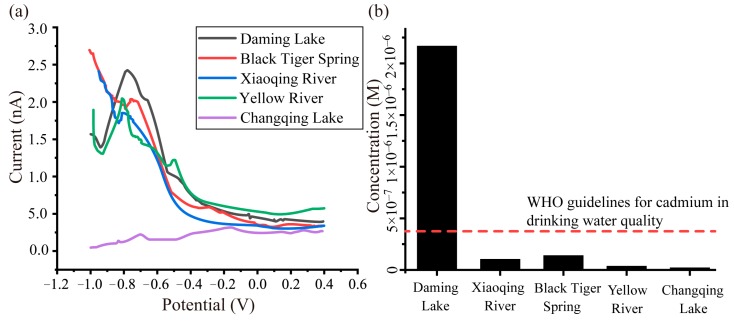
Water samples collected from several typical water sources around Jinan city (including the Daming Lake, the Black Tiger Spring, the Xiaoqing River, the Yellow River, and the Changqing Lake) were measured using the proposed carbon fiber electrode with nano-copper. (**a**) The DPV responses of the water samples. (**b**) The diagram for the Cd^2+^ concentration results of the water samples and their comparison with the suggested concentration given by the World Health Organization (WHO).

To verify the performance of our carbon fiber electrode sensor for Cd^2+^ detection, water samples collected from water sources around Jinan city, including the Daming Lake, the Black Tiger Spring, the Xioaqing River, the Yellow River, and the Changqing Lake. Figure 4 shows the DPV responses of these water samples with pH = 6.1. Their corresponding Cd^2+^ concentrations are shown in the diagram in Figure 4b, which was obtained from the curve fitting result in Section 3.5. According to the World Health Organization (WHO) guidelines for drinking water quality, cadmium ion concentration must be less than 0.005 mg/L (0.3 μM). Otherwise, the cadmium ion would be a threat to the health of the kidneys and other organs. The results revealed that the water in Daming Lake was not suitable for drinking directly. 

## 4. Conclusions 

In this study, a nano-copper-modified carbon fiber electrode was proposed and applied for Cd^2+^ inspection in drinking water. The detection limit, linear range, sensitivity and selectivity of the proposed sensor were justified. DPV technology was used to record the currents generated by the metal ions. The performance of the proposed carbon fiber electrode was also verified by measuring Cd^2+^ concentrations of water samples collected from water sources. The experiment results indicated that nano-copper deposition played a crucial rule in the improvement of the sensitivity of the electrode and the attraction ability for Cd^2+^ ions. 

Based on the results from this study, the next step for this sensor would be to integrate it into a portable device and apply it to the monitoring of the enrichment of heavy metal ions in the human body. The measuring results of Cd^2+^ concentrations could be sent to mobile phones by Bluetooth.

## Figures and Tables

**Table 1:** Comparison of Cd^2+^ sensing performance among our nano-copper-modified carbon fiber electrode and other electrodes in the literature.

Electrode Type	Detection Limit (nM)	Sensitivity (nA/nM)	Linear Range (nM)	Detection Method	Reference
GCE modified with CNT/poly pyrocatechol violet/bismuth	1.22	1.7	6.08~1820	ASV	[29]
N-doped carbon quantum dots-graphene oxide (NCQDs-Go)/GCE	45.3	0.16	0.67~683.6	ASV	[30]
GCE modified with gold nanoparticles(AuNPs)	0.045	/	0.0017~16.7	Colorimetri	[31]
Mo_6_S_9_ /GCE	0.61	260	3.04~912	DPASV	[32]
Nanocomposite based on nanographene	0.023	405	1.52~30.4	DPASV	[33]
Covalent anchoring of aryldiazonium salt	2.2	8.83 × 10^6^	25~500	SWASV	[34]
Bi doped mesoporous carbonxerogel/(GCE)	308	2.67 × 10^6^	6810~7540	SWASV	[35]
GCE modified with MWCNT	2.3	/	/	EIS	[36]
Bismuth nanorib bons(BiNRs) sensor	0.88	1.2 × 10^6^	6.08~304	DPASV	[37]
Au-Ph-AuNP-glutathione(GSH) electrode	0.01	9.17 × 10^7^	0.1~10	OSWV	[38]
Bismuthnanoparticle-porous/carbon paste electrode(CPE)	4.93	1.22 × 10^6^	6.08~608	SWASV	[39]
Carbon fibre electrode modified with nano-copper	10	3.7 × 10^8^	10~10^5^	DPV	This work
